# Multimorbidity of chronic diseases of lifestyle among South African adults

**DOI:** 10.11604/pamj.2021.38.332.15109

**Published:** 2021-04-06

**Authors:** Mukadas Oyeniran Akindele, Ushotanefe Useh

**Affiliations:** 1 Diseases of Lifestyle Niche Area, Faculty of Health Sciences, North West University, Mafikeng Campus, Mahikeng, South Africa,; 2 Department of Physiotherapy, Faculty of Allied Health Sciences, Bayero University, Kano State, Kano, Nigeria

**Keywords:** Hypertension, diabetes mellitus, chronic diseases, lifestyle

## Abstract

**Introduction:**

the prevalence of non-communicable and chronic diseases has been on the increase globally and has been a major factor responsible for high morbidity and mortality. Multimorbidity of the chronic diseases in low/medium income countries should be a major concern for public health practitioners because of the communicable diseases they also battle with. This study was carried out to determine the prevalence of multi-morbidity of chronic diseases of lifestyle (CDL) among adult South Africans.

**Methods:**

we employed General House Survey (GHS) data released by Statistics South Africa (Stats SA) in December 2015 with a response rate was 90.5%. Data on diabetes mellitus, high cholesterol, stroke, heart attack/myocardial infarction, hypertension were analysed using descriptive and inferential statistics.

**Results:**

the outcome of this secondary data analysis showed that about 16.3% of South Africans respondents aged 18 years and above had a single/or multiple CDL. The majority of the respondents with CDL were females (10.9%), older adults (9.4%), married (8.5%), of the black racial extract (11.9%), and reside in urban areas (3.4%). Also, high blood pressure (13.7%) was the most reported prevalent chronic disease while heart attack (1.1%) was the least reported chronic disease. The outcome of regression analysis after adjustment showed that gender (OR=0.56, CI=0.53-0.60, p<0.001), age [middle-aged adult (R=0.003, CI=0.003-0.004, p=0.001), older adult (R=0.25, CI=0.23-0.26, p=0.001)] and marital status [divorced (OR=1.55, CI=1.44-1.67, p<0.001), separated (OR=1.71, CI=1.46-2.00, p=0.001), single (R=1.88, CI=1.71-2.07, p=0.001)] were among the correlates of chronic diseases of lifestyle for the 1^st^ step of the adjustment. However, White population group (OR=1.17, CI=0.96-1.41, p=0.113), residing in farm settlement (OR=0.99, CI=0.84-1.16, p=0.910) and all the sub-scales of educational status were not correlates of the CDLs for the 2^nd^ adjustment of regression analysis.

**Conclusion:**

the findings of the study suggest that CDL is endemic among the South African population and that the most susceptible groups are the older adults, females, the married, the uneducated and the coloured individuals.

## Introduction

Public health and other health care practitioners are facing the challenges that arise from chronic diseases of lifestyle. Chronic diseases are the leading cause of death globally, and the prevalence is higher in low- and medium-income countries [1]. In 2011, WHO reported that about 80% of deaths that occurred in low- and medium-income countries result from chronic diseases [2]. It has also been projected that by the year 2020, chronic diseases like stroke will account for 75% of deaths, diabetes will account for 70% death and ischaemic heart disease will account for 71% deaths in low/medium income countries [3]. According to WHO [4], the African continent, comprising mainly low- and middle-income countries, has been projected to witness and experience the largest increase in the death rates from chronic diseases. In sub-Saharan Africa, it is reported that at least 69% of death observed on the African continent, results from chronic diseases along with communicable diseases [5]. Furthermore, it has also been documented that about 79% of all deaths attributable to chronic diseases globally occur in developing countries [6]. A few factors attributed to the increase in the burden of chronic diseases in Africa are urbanization, globalization, acculturation, changes in lifestyle as well as an increase in life expectancy [4]. In South Africa, 20% of deaths between the age of 35 and 64 years are attributed to chronic diseases of lifestyle [7]. The predisposing risk-factors for chronic diseases of lifestyle among South African in both rural and urban areas are physical inactivity, self-reported hypertension, tobacco smoking, and obesity [8].

The presence of two or more Chronic Diseases of Lifestyle (CDL) in people has been a challenge to healthcare professionals and public health practitioners [9]. The prevalence of the presence of two or more CDL in high-income countries has been documented despite access to good health care delivery system [10]. The terms co-morbidity and multimorbidity are two most commonly used to describe distribution/co-occurrence of diseases in an individual. Feinstein [11] introduced the term comorbidity and defined it as the combination of additional diseases beyond certain indices while multimorbidity refers to the co-occurrence of diseases in the same individual without any reference to certain indices [12]. The adverse effects of co-occurrence of CDL have been reported in the literature and their prevalence seems to be age-dependent [13,14]. About 85% of Americans aged 65 years and above have co-occurrence of CDL as against 23% amongst children [15]. Poor outcomes of health as a result of CDL include quality of life [13], mortality [16], disability [17] and adverse drug reactions from polypharmacy [18]. The US Department of Health and Human Services (HHS) developed a strategic framework to reduce the rising tide of the debilitating effects of multiple chronic conditions in the US [19]; which outlines the goals, objectives, and strategies to address the clinical and public health system changes in order to improve the health of the Americans [19]. Reports abound on the prevalence and descriptive epidemiology of individual with CDL in high- and low/medium-income countries such as Australia [20], Canada [21], the Netherlands [22], Sweden [23], Germany [24] and the United States [25]. However, there is a dearth of evidence on the multimorbidity prevalence of these diseases, especially in sub-Saharan Africa. This secondary data analysis was aimed at determining the prevalence of multimorbidity of CDL among adult South Africans.

## Methods

The data from the General House Survey (GHS) (2015) conducted, collected and released by Statistics South Africa (Stats SA) from January to December, 2015 was used for this study [26]. The GHS is an annual household survey conducted by Stats SA since 2002 covers all private households in all provinces in South Africa, excluding those living in students´ hostels, old-age homes, hospitals, prisons and military barracks. The areas covered by the GHS were education, health and social development, housing, household access to services and facilities, food security, and agriculture. The GHS national response rate was 90.5%. The section on ‘Health and General Functioning´ where the participants were asked if they had ever been told by a doctor/nurse/ or other health care worker at a clinic/hospital/private practice to have, most importantly, asthma, diabetes mellitus, cancer, HIV and AIDS, hypertension, arthritis, stroke, heart attack/myocardial infarction, tuberculosis, mental illness, epilepsy, meningitis and sinusitis, pneumonia, bronchitis, high cholesterol and osteoporosis among others. Multimorbidity was defined as the presence of two or more of these chronic conditions [27]. Sociodemographic variables extracted were age, gender, marital status, educational status, population group, geography type (urban or rural) and province residing. Data on diabetes mellitus, high cholesterol, stroke, heart attack/myocardial infarction, hypertension was analyzed for this study.

**Data source**: the data from the General House Survey (GHS) (2015) conducted, collected and released by Statistics South Africa (Stats SA) from January to December, 2015 was used for this study [26].

**Data analysis**: the data on diabetes mellitus, high cholesterol, stroke, heart attack/myocardial infarction, hypertension in adults aged 18 years and above were analyzed. The age range was categorised into young adult (18-35yr), middle-aged adult (36-65yr) and older adult (>66 yr) for ease of analysis. The data were presented using frequency and percentage to determine the prevalence of CDL in 2015. Chi-Square goodness of fit was used to determine the differences in the proportion of categorical variables. The dependent variable was the presence of NCDs while independent variables were age, gender, race, province, marital status, educational status, provincial residing and geographic type (urban, rural and semi-urban). A crude binary logistic regression was used to determine the correlates of the prevalence of CDL which was followed by adjustment of the sociodemographic variables using the presence of CDL as a dependent variable while age, gender and marital status were entered as independent variables at first step and province, racial group, geographical placement and educational level were entered as independent variables for the second step. Assumptions for logistic regression were checked for model´s fit (Omnibus test; Hosmer and Lemeshow test) and multicollinearity (Cook´s distance). The level of significance was set at 0.05.

## Results

**Figure 1:**
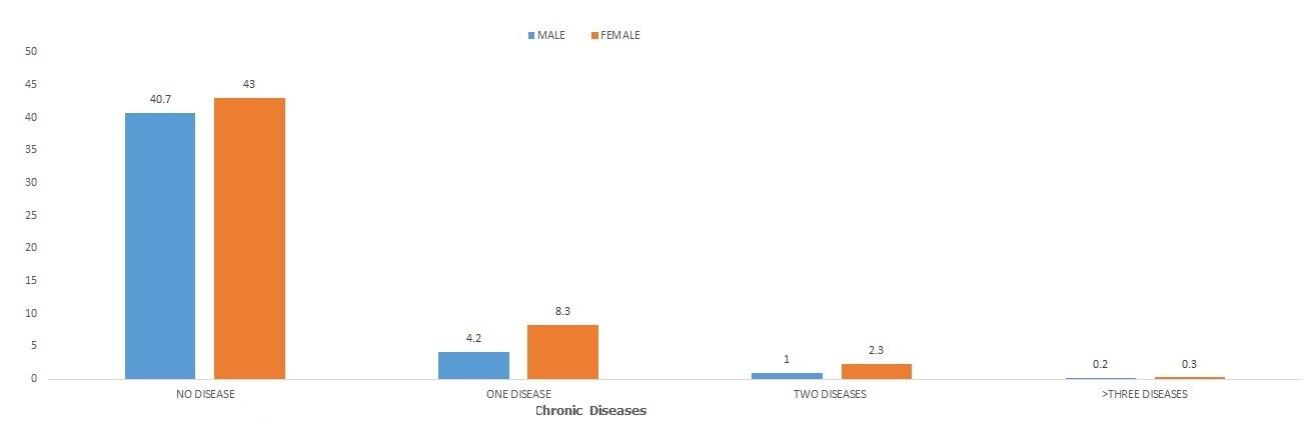
2015 GHS gender prevalence of chronic diseases

**2015 GHS sociodemographic details of South Africans aged >18 years**: in 2015, 47701 South Africans from 18 years and above participated in the general house survey (GHS) of which 46.1% were males while 53.9% were females. About 16.3% of the respondents reported to have presented with one or more CDL of which 5.4% were males while 10.9% were females as shown in Table 1. About 12.5% of the respondents had presented with one chronic disease, 3.3% had two chronic diseases and 0.5% had three or more chronic diseases as shown in Figure 1.

**Table 1:** 2015 GHS sociodemographic details of South Africans aged >18 years

Characteristics	Total (n=47701)	Male 21985(46.1%)	Female 25716(53.9%)
**Age**			
Young adult	22610	11059	11551
Middle-aged	16079	7318	8761
**Adult**			
Older Adult	9012	3608	5404
**No Disease**	39939(83.7)	19407 (40.7)	20532(43.0)
**Yes Disease**	7762(16.3)	2578(5.4)	5184(10.9)

**2015 GHS sociodemographic distribution of number of co-morbidity**: co-morbidities were found to be significantly associated with gender, age, marital status, highest educational attainment, population group, geographical type by province (Table 2). Those with the largest proportions with at-least one CDL were females (one CDL= 8.3%; two CDL= 2.3%, ≥ three CDL= 0.3%), older adults (one CDL= 6.8%; two CDL= 2.3%, ≥ three CDL= 0.3%), married respondents (one CDL= 6.6%; two CDL= 1.7%, ≥ three CDL= 0.2%), secondary educational attainment (one CDL= 5.7%; two CDL= 1.5%, ≥ three CDL= 0.2%), African/black residents (one CDL= 9.2%; two CDL= 2.4%, ≥ three CDL= 0.3%) and urban residents (one CDL= 8.5%; two CDL= 2.3%, ≥ three CDL= 0.3%). However, among the provinces, Gauteng had the largest proportion of respondents with one and two CDL, while Limpopo had the largest proportion of respondents with three or more CDL.

**Table 2:** 2015 GHS sociodemographic distribution of number of co-morbidity

	Total n(%)	No Disease n(%)	1 Disease n(%)	2 Diseases n(%)	>3 Diseases n(%)
**Gender**					
Male	21985(46.1)	19407(40.7)	2001(4.2)	500(1.0)	77(0.2)
Female	25716(53.9)	20532(43.0)	3966(**8.3**)	1088(**2.3**)	130(**0.3**)
**Age**					
Young Adult	22610(47.4)	22124(46.4)	428(0.9)	29(0.1)	29(0.1)
Middle-aged	16079(33.7)	13252(27.8)	2317(4.9)	465(1.0)	45(0.1)
Older Adult	9012(18.9)	4563(9.6)	3222(**6.8**)	1094(**2.3**)	133(**0.3**)
**Marital Status**					
Married	19758(41.4)	15717(32.9)	3131(**6.6**)	806(**1.7**)	104(**0.2**)
Divorced	1017(2.1)	695(1.5)	249(0.5)	69(0.1)	4(0.0)
Separated	444(0.9)	329(0.7)	89(0.2)	24(0.1)	2(0.0)
Widowed	3845(8.1)	1971(4.1)	1367(2.9)	461(1.0)	46(0.1)
Single	22637(47.5)	21227(44.5)	1131(2.4)	228(0.5)	51(0.1)
**Educational Status**					
No Formal Schooling	3611(7.6)	2438(5.1)	888(1.9)	266(0.6)	19(0.0)
Primary	7665(16.1)	5453(11.4)	1692(3.5)	466(1.0)	54(0.1)
Secondary	30218(63.3)	26711(56.0)	2706(**5.7**)	693(**1.5**)	108(**0.2**)
Tertiary	5992(12.6)	5159(10.8)	650(1.4)	158(0.3)	25(0.1)
Postgraduate	215(0.5)	178(0.4)	31(0.1)	5(0.0)	1(0.0)
**Population Group**					
African/Black	38025(79.7)	32383(67.9)	4379(**9.2**)	1124(**2.4**)	139(**0.3**)
Coloured	4652(9.8)	3617(7.6)	788(1.7)	215(0.5)	32(0.1)
Indian/Asian	1269(2.7)	1024(2.1)	148(0.3)	85(0.2)	12(0.0)
White	3755(7.9)	2915(6.1)	652(1.4)	164(0.3)	24(0.1)
**Geography Type**	31313(65.6)	26009(54.5)	4032(**8.5**)	1120(**2.3**)	152(**0.3**)
Urban	14487(30.4)	12297(25.8)	1720(3.6)	424(0.9)	46(0.1)
Traditional	1901(4.0	1633(3.4)	215(0.5)	44(0.1)	9(0.0)
**Province**					
Western Cape	5049(10.6)	4083(8.6)	752(1.6)	191(0.4)	23(0.0)
Eastern Cape	6139(12.9)	4966(10.4)	850(1.8)	289(0.6)	34(0.1)
Northern Cape	2211(4.6)	1697(3.6)	398(0.8)	101(0.2)	15(0.0)
Free State	2808(5.9)	2207(4.6)	455(1.0)	128(0.3)	18(0.0)
Kwazulu-Natal	8400(17.6)	7140(15.0)	925(1.9)	295(0.6)	40(0.1)
North West	3074(6.4)	2496(5.2)	469(1.0)	101(0.2)	8(0.0)
Gauteng	11413(23.9)	9738(20.4)	1302(**2.7**)	320(**0.7**)	53(0.1)
Mpumalanga	3667(7.7)	3140(6.6)	438(0.9)	77(0.2)	13(0.0)
Limpopo	4940(10.4)	4472(9.4)	378(0.8)	86(0.2)	4(**0.2**)

**Prevalence of chronic diseases by gender**: high blood pressure was the most prevalent (13.7%) with more females reporting having high blood pressure (9.5%) while heart attack recorded the least prevalent (1.1%). The association between sex and the occurrence of high blood pressure, diabetes mellitus and heart attack was significant (p>0.001), while the occurrence of stroke and cancer by sex was not significant (p>0.05) (Table 3).

**Table 3:** prevalence of chronic diseases by gender

Chronic diseases	Male n(%)	Female n(%)	P	Total n(%)
**High Blood Pressure**	2035 (4.3)	4520(9.5)	<0.001	6555 (13.7)
**Diabetes Mellitus**	805(1.7)	1407(2.9)	<0.001	2212(4.6)
**Stroke**	138(0.3)	171(0.4)	>0.05	309(0.6)
**Cancer**	107(0.2)	150(0.3)	>0.05	257(0.5)
**Heart Attack**	190(0.4)	327(0.7)	<0.001	517(1.1)

**Correlates of multimorbidity of chronic diseases of lifestyle**: logistic regression was used to determine the association of sociodemographic variables on reporting chronic diseases. The model had seven independent variables (age, gender, marital status, educational level, population group, province, geographical area). The full model of the independent variables was significant, χ^2^ (24, N=47701)= 11486.415, p= 0.001. Crude binary logistic regression reveals that gender (female), age (middle-aged and older adults), marital status (widowed and single), educational status (primary school completed), population group (Coloured and White), geography type (traditional and farms) and province (Northern Cape, Free State, Kwazulu-Natal, North West and Limpopo Provinces) were all correlates of diseases of lifestyle of all the seven independent variables. The strongest correlate of chronic diseases was age (older adult) (odds ratio of 32.108), indicating that older adults were over thirty two times more likely to have chronic diseases than those who did not have chronic diseases. Limpopo Province presented with an odds ratio of 0.440 (less than 1) (Table 4). The outcome of Step 1 of the adjustment showed that gender (OR= 0.56, CI= 0.53-0.60, p= 0.001), age [middle-aged adult(R= 0.003, CI= 0.003-0.004, p= 0.001), older adult (R= 0.25, CI= 0.23-0.26, p= 0.001)] and marital status [divorced (OR=1.55, CI= 1.44-1.67,<0.001)'> p< 0.001), separated (OR= 1.71, CI= 1.46-2.00, p= 0.001), single (R= 1.88, CI= 1.71-2.07, p= 0.001)] were among the correlates of chronic diseases of lifestyle for the 1^st^ step of the adjustment. However, White population group (OR= 1.17, CI= 0.96-1.41, p= 0.113), residing in farm settlement (OR= 0.99, CI= 0.84-1.16, p= 0.910) and all the sub-scales of educational status were not correlates of the CDLs for the 2^nd^ adjustment of regression analysis, as shown in Table 4.

**Table 4:** correlates of multimorbidity of chronic diseases of lifestyle

Variables	Bivariate Analysis (Crude)		Multivariate Analysis (Adjusted)			
			Step 1		Step 2	
	p-value	OR (95% CI)	p-value	COR(95% CI)	p-value	AOR(95% CI)
**Gender**						
Male	I					
Female	0.001*	1.83(1.73-1.95)	0.001*	0.56(0.53-0.60)	0.001*	0.56(0.51-0.58)
**Age**						
Young Adult	I					
Middle-Aged Adult	0.001*	7.71(6.96-8.56)	0.001*	0.03(0.03-0.04)	0.001*	0.03(0.03-0.04)
Older Adult	0.001*	32.11(28.69-35.93)	0.001*	0.25(0.23-0.26)	0.001*	0.24(0.23-0.26)
**Marital Status**						
Married	I					
Divorced	0.266	1.09(0.94-1.27)	0.001*	1.55(1.44-1.67)	0.001*	1.63(1.51-1.76)
Separated	0.314	0.89(0.70-1.21)	0.001*	1.71(1.46-2.00)	0.001*	1.77(1.51-2.08)
Widowed	0.001*	1.24(1.14-1.35)	0.003*	1.44(1.14-1.83)	0.003*	1.44(1.13-1.84)
Single	0.001*	0.61(0.57-0.66)	0.001*	1.88(1.71-2.07)	0.003*	2.02(1.83-2.23)
**Province**						
Western Cape	I					
Eastern Cape	0.743	0.98(0.86-1.11)			0.001*	2.27(1.95-2.66)
Northern Cape	0.001*	1.37(1.18-1.58)			0.001*	2.23(1.95-2.54)
Free State	0.003*	1.25(1.08-1.45)			0.001*	3.10(2.62-3.68)
KwaZulu-Natal	0.337	0.94(0.82-1.07)			0.001*	2.85(2.43-3.34)
North West	0.003*	1.25(1.08-1.45)			0.001*	2.13(1.87-2.43)
Gauteng	0.013*	0.86(0.77-0.97)			0.001*	2.85(2.45-3.31)
Mpumalanga	0.232	0.91(0.78-1.06)			0.001*	1.96(1.72-2.24)
Limpopo	0.001*	0.44(0.38-0.51)			0.001*	2.07(1.78-2.41)
**Population Group**						
Black/African	I					
Coloured	0.004*	1.18(1.06-1.32)			0.001*	1.31(1.18-1.46)
Indian/Asian	0.182	0.89(0.75-1.06)			0.001*	1.55(1.36-1.76)
White	0.001*	0.76(0.69-0.85)			0.113	1.17(0.96-1.41)
**Geographical Type**						
Urban	I					
Traditional Setting	0.001*	0.75(0.68-0.81)			0.001*	1.33(1.14-1.55)
Farms	0.001*	0.75(0.65-0.88)			0.910	0.99(0.84-1.16)
**Educational Status**						
No formal Schooling	I					
Primary School Completed	0.001*	1.24(1.12-1.37)			0.592	1.12(0.75-1.67)
Secondary School Completed	0.732	0.98(0.89-1.08)			0.108	1.38(0.93-2.05)
Tertiary Education	0.057	0.87(0.78-1.00)			0.643	1.10(0.74-1.62)
Postgraduate	0.592	0.89(0.60-1.34)			0.953	0.99(0.67-1.47)

## Discussion

This paper sought to determine the prevalence of co-occurrence of CDL among adult South Africans. It was revealed that about 16.3% of South Africans respondents aged 18 years and above had a single/or multiple CDL. The majority of the respondents with CDL were females, older adults, married, of the black racial group, and reside in urban areas. High blood pressure was also the most reported chronic disease while heart attack was the least reported one. Gender, age, marital status, educational status, geographic area and province residing were all correlates of chronic diseases of lifestyle. Age (older adult) was a major correlate while the province (Limpopo) was a least correlate of chronic diseases of lifestyle.

This study shows that about 16.3% of the GHS 2015 South African respondents had one or more CDL which is in conformity with previous studies from low/medium and high-income countries. About 21% of Americans had more than one chronic condition or multiple illnesses. This high prevalence reported by Vogeli *et al*. might be due to the fact that their study population also included children [28]. In a recent study by Wald *et al*. high prevalence of 49.8% was also reported. In their study, almost half (of non-institutionalised US civilian adults had at least one out of ten selected CDL while 25.5% had two or more CDL [29]. The Canadian study indicated that about 12.9% of adult aged >20 years had two or more chronic diseases and 3.9% had three or more in 2011/12 period [30]. Wu *et al*. reported that 50% of their participants amongst the adult population in China, aged 50 years and above, had one chronic condition while about 26.1% had two or more chronic conditions [31]. In a developing country of sub-Saharan Africa, multimorbidity prevalence of 38.8% was reported among Ghanaians patients aged 18-59 years attending a clinic [32] and 53.8% among adults aged >60 in Bangladesh [33] as against 3.8% in the current study. The low multimorbidity observed in our study might be due to the fact that the data for this study was obtained through interviews and not measured.

Multiple CDL has been shown to be more prevalent in persons with certain sociodemographic variables. We observed that respondents who were females, older adults, married, of the black racial group, and reside in urban areas had one or multiple CDL. Ashman and Beresovsky reported a contrary finding regarding gender prevalence of multiple CDL that more men than women visited physician office in 2009 in the US [34]. This finding might be due to the fact that Ashman and Beresovsky used the National Ambulatory Medical Care Survey (NAMCS) which was not a representative of the whole American populace [35]. Our data source was from GHS (2015) representing South Africans. However, our finding was in line with the study by Roberts *et al*. who reported that the prevalence of multiple CDL was more among females, older adults and urban residents than males, younger individuals and rural residents respectively [30]. Also, this finding is consistent with Marengoni *et al*. [35] systematic review where it was reported that aged individuals, women were more likely affected by multimorbidity of CDL than males. Furthermore, the prevalence of multimorbidity of CDL among Americans medical beneficiaries in 2010 was reported to be more among women, non-Hispanic black and Hispanic women and increased with age [36]. This is consistent with the outcome of this study. Increased life expectancy and access to quality healthcare in high-income countries are attributed to the higher prevalence of multimorbidity of CDL in older adults [37]. Also, genetic factors, living and working environments, life events, behavioural risk factors or the general risks associated with low socioeconomic status have been implicated as the reasons for the high prevalence of multimorbidity among the female gender [33].

High blood pressure was the most prevalent reported CDL by the respondents, while myocardial infarction was the least reported. These two conditions were more frequently reported by female respondents. Hypertension has been shown to be the most commonly reported CDL multimorbidity by Chinese elders [31]. Also, hypertension was the most prevalent among the American Medicare beneficiaries [36] and among the German elderly population [24]. Furthermore, a study from Ghana showed that hypertension (52.8%) was the most prevalent conditions among the 13 pre-selected chronic conditions [30]. These findings from middle- and high-income countries are consistent with that of the current study. In addition, a high prevalence of hypertension was earlier reported among adult South Africans [37]. The current finding may be attributed to technological transitioning of African countries which encourages sedentary lifestyles, as well as urbanization and westernized acculturation. This finding is expected to alert the South-African public-health physicians to be more focused on addressing the menace of CDL and be aware that management should not be tailored to one chronic disease but multi-targeted.

Gender, age, marital status, educational status, geographic area and province residing were all correlates and risk factors of multimorbidity of CDL among our respondents. Age (older adult) was a major predictor while the province (Limpopo) was at least predictor of chronic diseases of lifestyle. It has been reported in the literature that these sociodemographic factors are predictors of CDL and are associated with their multimorbidity. Among the Canadians, Agborsangaya *et al*. reported age, gender, income and family structure were predictors of chronic disease multimorbidity (>18 years) [38]. Alaba and Chola also reported that gender and age were predictors of chronic disease multimorbidity using South Africa National Income Dynamic Survey (SA-NIDS) of 2006 [39]. Irrespective of the sources of data, Alaba and Chola [39] and our study underscore the need for redirection of efforts in the prevention and management of CDL bearing in mind the enormous economic and health burden that multimorbidity poses.

Consistent with previous studies, we found out that older adulthood was a strong correlate of chronic disease multimorbidity. South Africans above the age of 65years were more susceptible to chronic disease multimorbidity. This trend had been reported earlier on in high-income countries [20] which might be due to increase in the ageing population and longevity as well as an increase in the prevalence of CDL [27,40]. Our study also showed that Limpopo Province residents were less likely to have chronic disease multimorbidity which is supported by the study of Weimann *et al*. [41] that all the five districts in Limpopo province had a lower multimorbidity prevalence when compared with other provinces after adjusting for age which may be due to the fact that most of the residents were socioeconomically disadvantaged [41].

## Conclusion

The findings in the study suggest that CDL is endemic in the South African population and that the most susceptible groups are the older adults, females, the married, the uneducated and the coloured individuals. These findings are expected to be the beam of the searchlight for the South African healthcare professional as to the groups to target and give focused attention in a bid to reduce the prevalence of CDL and their multimorbidity. Since South Africa is a country with people of diverse cultural affiliations, preventive and therapeutic strategies could be tailored towards socio-cultural beliefs that encourage multimorbidity.

### What is known about this topic

- Evidence of multimorbidity of chronic diseases of lifestyle in high-income countries;
- Increase in the prevalence of chronic diseases of lifestyle in sub-Saharan Africa.

### What this study adds

- Multimorbidity of chronic diseases of lifestyle among South African adults;
- More common among >18 years female and South Africans within older adults age group (>66 years) had a high correlation with CDLs;
- High blood pressure was the most reported condition.
